# Development and Clinical Validation of the LymphMonitor Technology to Quantitatively Assess Lymphatic Function

**DOI:** 10.3390/diagnostics11101873

**Published:** 2021-10-12

**Authors:** Anna Polomska, Epameinondas Gousopoulos, Daniel Fehr, Andreas Bachmann, Mathias Bonmarin, Michael Detmar, Nicole Lindenblatt

**Affiliations:** 1 Swiss Federal Institute of Technology (ETH Zürich), Institute of Pharmaceutical Sciences, Vladimir-Prelog Weg 3, 8093 Zurich, Switzerland; anna.kedracka@pharma.ethz.ch; 2 Department of Plastic and Hand Surgery, University Hospital Zurich, Rämistrasse 100, 8091 Zurich, Switzerland; epameinondas.gousopoulos@usz.ch (E.G.); Nicole.Lindenblatt@usz.ch (N.L.); 3 Zurich University of Applied Sciences (ZHAW), Institute of Computational Physics, Technikumstrasse 9, 8401 Winterthur, Switzerland; daniel.fehr@zhaw.ch (D.F.); andreas.bachmann@zhaw.ch (A.B.); mathias.bonmarin@zhaw.ch (M.B.)

**Keywords:** lymphatic system, lymphedema, mobile health, lymphatic function monitoring, lymphography, indocyanine green

## Abstract

Current diagnostic methods for evaluating the functionality of the lymphatic vascular system usually do not provide quantitative data and suffer from many limitations including high costs, complexity, and the need to perform them in hospital settings. In this work, we present a quantitative, simple outpatient technology named LymphMonitor to quantitatively assess lymphatic function. This method is based on the painless injection of the lymphatic-specific near-infrared fluorescent tracer indocyanine green complexed with human serum albumin, using MicronJet600^TM^ microneedles, and monitoring the disappearance of the fluorescence signal at the injection site over time using a portable detection device named LymphMeter. This technology was investigated in 10 patients with unilateral leg or arm lymphedema. After injection of a tracer solution into each limb, the signal was measured over 3 h and the area under the normalized clearance curve was calculated to quantify the lymphatic function. A statistically significant difference in lymphatic clearance in the healthy versus the lymphedema extremities was found, based on the obtained area under curves of the normalized clearance curves. This study provides the first evidence that the LymphMonitor technology has the potential to diagnose and monitor the lymphatic function in patients.

## 1. Introduction

The lymphatic system plays a pivotal role in immune surveillance, absorption of dietary lipids in the intestine, and tissue fluid homeostasis [1,2]. Thus, alterations of lymphatic system function have implications in a number of diseases, including lymphedema [3], advanced-stage lipedema [4,5,6], venous insufficiencies [7], impaired wound healing, and chronic inflammatory diseases [8,9,10]. Among those, lymphedema, resulting from fluid accumulation in the tissues, leads to the development of chronic, often disabling, and disfiguring swelling of the affected body part. While primary lymphedema is rare and caused by somatic mutations, secondary lymphedema can develop after oncologic surgery (where usually lymphadectonomy is part of the treatment regime), radiotherapy, infection (lymphangitis or parasitic infection), or other major injuries [11,12,13,14].

Quantitative evaluation of the lymphatic function is useful for early diagnosis, accurate staging, and evaluation of the treatment of lymphatic insufficiencies, such as lymphedema. [15,16,17]. Some quantitative approaches measuring the rate of disappearance of a lymphatic-specific radioactively labeled tracer at the injection site—which reflects lymphatic vessel functionality—have been used in clinics. However, they require stationary and expensive scintigraphy cameras which drastically hampers the widespread applicability of these methods [18,19,20]. Thus, a technology that enables simple and quantitative monitoring of lymphatic vessel function in outpatient settings remains an unmet medical need. The use of near-infrared (NIR) fluorescent tracers, such as indocyanine green (ICG), allows the elimination of the need for radioactivity and the associated risk of ionizing radiation, as well as the use of bulky instrumentation [20]. Quantitative approaches for evaluating lymph transportation capacity by measuring ICG velocity [21,22,23] or pumping pressure [20,24,25,26,27] using fluorescence cameras have been applied in clinics. We and others have developed techniques for the quantitative measurement of lymphatic vessel drainage that are based on monitoring the decay of a fluorescence signal after tissue injection of lymphatic-specific NIR fluorescent tracers in animal models [27,28,29,30,31]. In a recent study, which laid the foundation for the current clinical validation, we developed a new, three-pronged approach for measuring lymphatic function in vivo [27]. We produced a new formulation of ICG containing the surfactant polyoxyl 15 hydroxystearate (brand name: Kolliphor HS15) that helped to overcome the physicochemical limitations of ICG, such as aggregation and self-quenching in purely aqueous solutions [28,30,32,33,34]. The ICG-Kolliphor HS15 solution was administered intradermally via a microneedle-based injection device with hollow microneedles, named MicronJet600 (Nanopass Ltd., Ness Ziona, Israel). We then used a custom-made handheld NIR detection device named LymphMeter 1.0 for the simple monitoring of the NIR signal at the injection site. This custom-made device is portable, equipped with an external power source and therefore suited for use outside of a hospital setting, in contrast to existing imaging technologies. Using this device, we were able to quantitatively determine the lymphatic vessel function in pig skin, based on the clearance dynamics of the tracer from the injection site [27]. 

In the present study, we investigated a new formulation of ICG pre-complexed with human serum albumin (HSA) in animals and in humans in combination with a LymphMeter measuring device and hollow MicronJet600 microneedles. The aim of the animal studies in mice and pigs was to validate the selected ratio of ICG-HSA and determine the required measurement period for the human study. The aim of the following investigator-initiated proof-of-concept human study in 10 patients suffering from unilateral lymphedema was to evaluate whether using this method, named LymphMonitor 1.0, allows for distinguishing normal and impaired lymphatic function in healthy and lymphedematous extremities in humans. Overall, the study provides the first evidence that the quantitative evaluation of lymphatic clearance of intradermally injected HSA-ICG (LymphMonitor technology) has the potential to evaluate the lymphatic function in patients with lymphatic insufficiencies.

## 2. Materials and Methods

### 2.1. Chemicals and Stability Tests

Commercially available, clinical-grade ICG and HSA were used in this study: VERDYE, (Diagnostic Green GmbH, Aschheim-Dornach, Germany) and Albumin CSL 5% and 20% (CSL Behring, Marburg, Germany). 

### 2.2. In Vivo Clearance in Animals 

Mice were kept under specific pathogen-free conditions until imaging. FVB mice were bred in-house. K14-sVEGFR-3-Fc transgenic mice were kindly provided by Dr. Kari Alitalo, University of Helsinki, Finland [29,35]. Mouse experiments were performed in accordance with an animal protocol (ZH212/16) approved by the Cantonal Veterinary Office Zurich, Switzerland. An IVIS Spectrum (Xenogen, Caliper Life Sciences, Hopkinton, MA, USA) imaging system was used for the in vivo lymphatic clearance assay. Male mice, 10–12-weeks-old, were used for the assays (FVB WT or K14-VEGFR3-Fc transgenic). Detailed protocols for this assay are described elsewhere [27,29,34,35]. In brief, mice under 2% isoflurane anesthesia were injected intradermally in the ears with 3 µL of a freshly prepared solution containing ICG (0.0025 or 0.005 mg/mL in 5% and 20% HSA or in water using 29-G insulin syringes (Terumo). The fluorescence signal in the ears was monitored at pre-determined timepoints (0, 1, 2, 4, 6 and 24 h). Measurements were performed in isoflurane anesthetized mice. Between the measurements, the animals were allowed to move without any restriction. The signal was measured using the following settings: λ_ex_/λ_em_ = 745/800 nm, exposure time of 1.5 or 2 s, small binning, field of view 6.6 cm × 6.6 cm. The images were analyzed by drawing a region of interest (ROI) around the administration site, and the average fluorescence intensity was measured for each timepoint using Living Image Software (Caliper Life Sciences). After subtracting the background signals, the values were normalized to the time 0 measurement and plotted against time. A mono-exponential decay function was fitted to the obtained data and the dermal elimination half-lives were calculated using GraphPad Prism 7 software (constrain parameters for fitted curve: at time 0 h, the value is equal to 1, the plateau value is 0).

The pig experiment was performed in the Centre National de Biologie Expérimentale, Institute National de la Recherche Scientifique (INRS), Laval, Canada. Procedures involving the care and the use of pigs in this study were reviewed and approved by the INRS Institutional Animal Care and Use Committee (performed according to the Canadian Council on Animal Care (CCAC) guidelines and policies (study protocol number: 1711-01)). Throughout the experiment, the health and well-being of the pigs were closely monitored by a veterinarian. One 4-week-old female domestic pig was obtained from local Canadian farmer. Prior to starting any procedures, the animal was acclimatized for one week in the facility. 

The pig was placed on the restrain sling for the injections. Three intradermal injections of 50 µL of freshly prepared ICG (0.0025 mg/mL)-HSA (5%) were performed on each side of the flank using MicronJet600TM (NanoPass Technologies Ltd., Ness Ziona, Israel) microneedles attached to 1 mL BD Luer-Lok syringes [36]. After the injection, a circular area (3 cm diameter) around the injection site was marked. For each measurement, the pig was placed on the sling and the LymphMeter detection device was moved slightly around the marked injection area to localize the region with the highest signal intensity. Eight to eleven measurements were recorded, and the three highest values were used for data analysis. Between the measurements it was allowed to move without any restrictions. 

### 2.3. Human Study

The clinical study was approved by the Zurich Cantonal Ethics Committee (BASEC number: 2018-01823) and Swiss Agency of Therapeutic Products (Swissmedic, study number: 2020DR1060). The trial was conducted according to the principles of the Declaration of Helsinki and Good Clinical Practice standards. The study was registered in the Swiss National Clinical Trial Registry at kofam.ch (SNCTP000003646) and at ClinicalTrials.gov (NCT04393168).

#### 2.3.1. Study Design and Endpoints

The study was performed at the Division of Plastic and Hand Surgery of the University Hospital Zurich, Zurich, Switzerland. The study was a monocentric, interventional, intra-individual comparison to assess whether the LymphMonitor method allows for valid assessment of lymphatic function in humans in the context of secondary lymphedema. The primary objective of the study was to investigate the feasibility of the method to assess lymphatic function in 10 arm or leg lymphedema patients (diseased extremity versus healthy extremity). The secondary objective was to establish a correlation of clearance parameters with the extent of the swelling in lymphedema patients. Safety objectives included the evaluation of skin reactions after administration of the tracer solution, the assessment of potential allergic reactions and intolerances, as well as the assessment and reporting of all adverse effects (expected and unexpected) for the full period of the study.

#### 2.3.2. Participants

A total of ten patients (7 female and 3 male) were recruited for the study. A detailed list of inclusion and exclusion criteria is provided in Table 1. 

The description of the patient population is shown in Table 2, together with the measured volume of the extremities. The volumes of lymphedema extremities were on average 21.9 ± 12.7% higher compared to contralateral, non-affected arms and legs.

#### 2.3.3. Visits and Intervention

The study consisted of two visits: screening visit and interventional visit. Before the screening visit, each patient obtained and signed informed consent after the nature and possible consequences of the studies were explained in detail.

##### Screening Visit

During the first visit, the patients’ eligibility for the study was determined based on inclusion/exclusion criteria. A basic physical examination was performed (blood pressure and temperature). Moreover, the information about lymphedema-related medical history was collected, which included origin of lymphedema (i.e., cancer, infection, or operation-related lymphedema), suffered symptoms (e.g., pain, heaviness, itchiness), and the type and frequency of applied lymphedema therapies.

##### Investigational Visit

During the second, interventional visit, the patients had the volumes of their healthy and lymphedema limbs measured following an established standard operating procedure (SOP) of the Department of Physiotherapy of the University Hospital Zurich (USZ). The measurements were performed by a physiotherapist specialized in lymphological therapy. Briefly, sequential perimeters were measured every 4 cm starting from the level of the wrist (from processus styloideus ulnae) or ankle (from malleolus lateralis) until the level of the axilla (for arm) or groin (for leg). The volume was calculated using the truncated cone formula [38] using a standardized interface. After that, the ICG (0.0025 mg/mL)-HSA (5%) solution was prepared (as described in Section 2.3.4). For injections of ICG-HSA and measurements, the patients were placed in the supine or sitting position. The injections were allocated in the visually most swollen region of the arm or the leg and performed in the symmetric area of the contralateral non-swollen arm (forearm: in the dorsal or ventral part)/lower leg (anterior or posterior). Injection of 50 μL of the ICG-HSA solution was performed using MicronJet600 microneedles according to the manufacturer’s instructions. The injection sites were marked with a non-allergenic marker. Moreover, the arm/leg was photographed after the injection and the circumference at the injection site in both the healthy and the lymphedemous arm/leg was measured using measuring tape.

**Figure 1:**
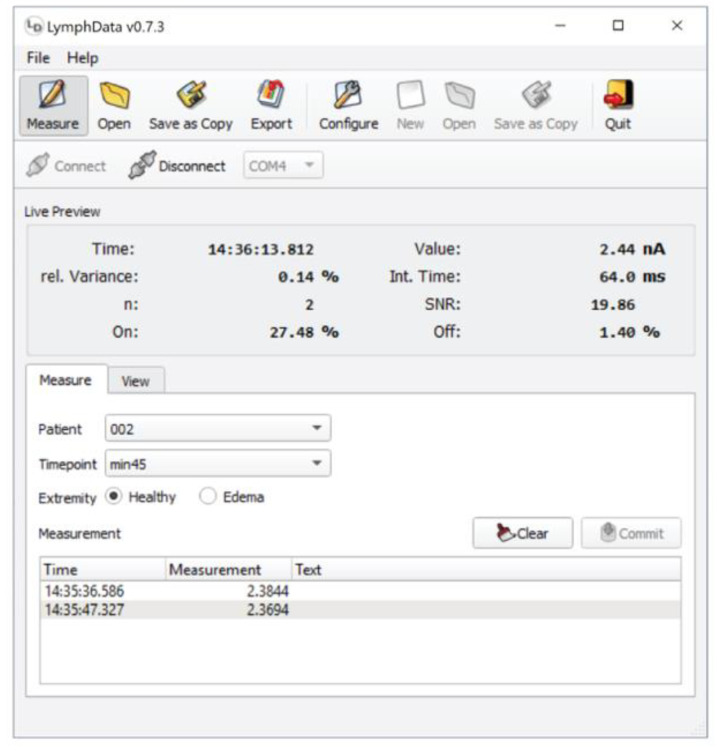
Custom LymphData software used in the clinical study to record the measurements and export them in the format suitable for direct export into eCRF. The LymphMeter is connected to the LymphData software via Bluetooth.

The time and the location of the injection was recorded in electronic Case Report Form (eCRF, secuTrial). Following the injection, the fluorescence signal at the injection site was measured with the LymphMeter (every 15 min during the first hour, and every 30 min thereafter for the total duration of 3 h). For each timepoint, six measurements per extremity were performed. Custom LymphData software (Figure 1, described in the following paragraph) connected via Bluetooth with the LymphMeter device was used to record, save, and export the data in a format that was compatible with the secuTrial data capture system.

Between the measurements, the patients were asked to stay mainly in a comfortable sitting position and were allowed to perform simple activities that do not require intense arm or leg movements. For example, for arm measurements the patient were allowed to watch a movie or read a book, but not to knit or sew. For leg measurements, the patients were allowed to go to the bathroom but not use the stairs, do any jogging, or walk long distances.

#### 2.3.4. Preparation of ICG-HSA

The ICG-HSA solutions containing 0.0025 mg/mL ICG in 5% HSA were prepared in a dilution/reconstitution process, by firstly dissolving the content of the VERDYE vial (25 mg) in sterile water for injection followed by dilution of 1 mL of the resulting solution in 500 mL of commercial 5% HSA. The final solution was used within one hour from the preparation. One preparation was performed for each patient and the remaining solutions were discarded.

#### 2.3.5. LymphMeter and LymphData Software

The LymphMeter device has been already described in detail in our recent publication [27]. For the purpose of the current study, the LymphData software was developed to facilitate recording measurements and the import of the data to the secuTrial-based electronic Case Report Form (eCRF) (Figure 1). LymphData supports Windows, MacOS, or Linux based systems. It is connected via Bluetooth to LymphMeter 1.0. When a measurement is triggered by pressing the button on the side of LymphMeter 1.0, LymphData receives and saves the measurement from LymphMeter 1.0 in real time and automatically associates it with metadata, such as the time and date of the measurement (timestamp) and pre-defined parameters such as time point, patient ID and the measured extremity code (healthy or lymphedema). The aggregated data is saved in LymphData’s internal storage system. The measurements can then be exported to files with a particular comma-separated file format that the eCRF data management system can interpret. Therefore, the user can transfer the measurements directly into the eCRF in a semi-automated manner that assures the integrity of the measurements. Moreover, the operating conditions of the LymphMeter 1.0 (i.e., the laser diode integration time or signal-to-background ratio) are also reported to the LymphData software, where they are displayed and saved in the internal storage system together with the actual measurement. If proper operation of the LymphMeter 1.0 is questioned during evaluation of the measurements at a later stage of the study, these recordings can be consulted to investigate whether the device was working properly and was correctly operated. Thanks to its internal database, LymphData can handle prolonged studies over several weeks, interrupted by restarts of the host system, forced closing, power outages, or similar incidences, without any data loss.

#### 2.3.6. Data and Statistical Analysis of the Clinical Study

From six measurements for each timepoint, the average fluorescence intensity was calculated. The average intensity values were normalized to the value at timepoint 0 h (immediately after injection) and the normalized data were plotted against time to generate clearance curves. The AUC was calculated using the trapezoidal method. Normality of the obtained AUC datasets for healthy and lymphedema extremities were tested using D’Agostino&Pearson normality test. The parameters in healthy and affected extremities were statistically compared using the parametric paired Student’s t-test (mean comparison); 95% confidence intervals for the mean were calculated. Data are presented as mean ± standard deviation (S.D.).

## 3. Results and Discussion

### 3.1. ICG-HSA Can be Used to Quantitatively Assess Lymphatic Function in Mice

The aim of the first part of the study was to determine whether the selected ICG (0.0025 mg/mL) solution in a sterile, commercially available 5% HSA can be used for the quantitative assessment of lymphatic clearance in vivo. These concentrations were based on the following criteria: the selected ICG concentration (in 5% HSA) lies in the linear range of ICG concentration—fluorescence intensity curve (measured in vitro, Supplementary Methods and and Figure S1a) and ICG solutions in commercially available 5% HSA were easy to inject in preliminary experiments in mice and did not leak from the intradermal injection sites, as opposed to 20% HSA solutions. We also confirmed improved in vitro stability of the ICG in 5% HSA solution in contrast to purely aqueous solution, by measuring fluorescence intensity over time. While the fluorescence intensity of ICG in water solution decreased by around 60% after 24 h, indicating dye degradation, the fluorescence intensity of ICG-HSA remained stable within this time (Supplementary Figure S1b). The longer stability of ICG-HSA has positive implications for its clinical use as it increases the in-use time.

To determine whether the selected ICG-HSA formulation can be used for the quantitative assessment of lymphatic clearance, we compared the lymphatic clearance in ears of wildtype (WT) mice and of K14-VEGFR3-Fc transgenic mice that lack dermal lymphatic vasculature. Briefly, we injected 3 μL of the solution into the ears of mice and followed the fluorescence signal intensity over time using IVIS Spectrum Imaging System.

**Figure 2:**
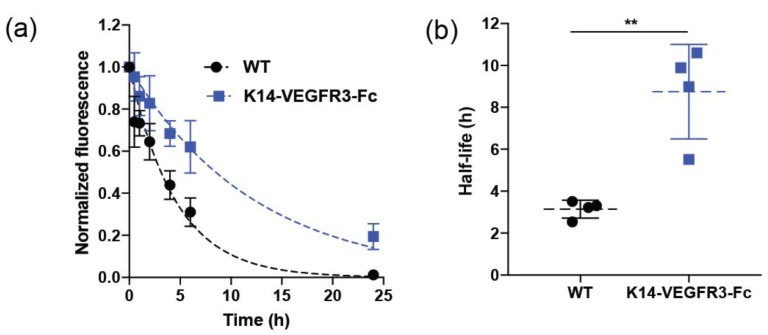
Quantification of dermal lymphatic drainage after bolus intradermal administration of 3 µL of ICG (0.0025 mg/mL) in 5% HSA in the ears of WT and K14-VEFGR3-Fc mice lacking a dermal lymphatic vasculature. (**a**) Normalized fluorescence intensity over time and fitting of mono-exponential decay function in WT and K14-VEGFR3-Fc (*n* = 4 mice per group) mice. (**b**) Quantification of dermal elimination half-lives in WT and K14-VEFGR3-Fc mice from fitted curves. Data are shown as mean ± S.D and compared by Student’s t-test; ** *p* < 0.01.

Figure 2a,b show the fluorescence intensity over time and fitting of mono-exponential decay function in wild type and K14-VEGFR3-Fc mice and the calculated half-life of clearance, respectively. The calculated half-life of clearance was significantly longer in K14-VEGFR3-Fc mice than in WT mice (mean ± S.D: 8.7 ± 2.3 h vs. 3.1 ± 0.4 h respectively, ** *p* < 0.01, Student’s *t*-test. Figure 2b). These values are comparable to those previously obtained using ICG-Kolliphor HS15 (mean ± S.D: 9.6 ± 0.4 h and 2.6 ± 0.8 h for K14-VEGFR3-Fc mice and WT mice, respectively) [27]. Overall, these results indicate that the ICG-HSA formulation can be used for the quantitative assessment of lymphatic function in vivo.

### 3.2. In Vivo Validation of ICG-HSA in Pigs Using the LymphMeter Device

**Figure 3:**
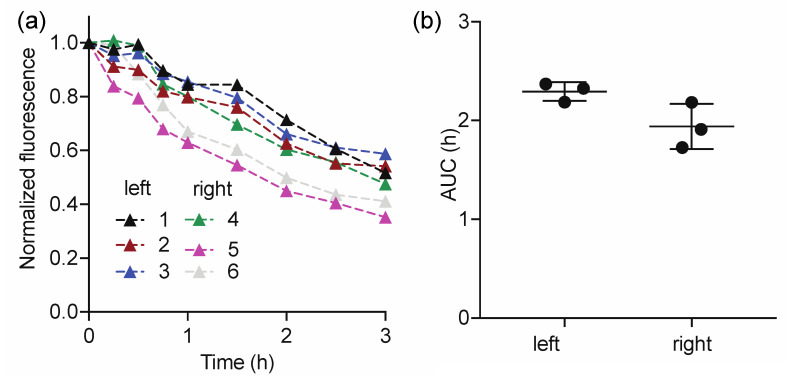
Lymphatic clearance measured by the LymphMeter 1.0 in the skin of the pig’s flank after bolus intradermal administration of 50 µL of ICG (0.0025 mg/mL) in a solution of 5% HSA, using MicronJet600^TM^ microneedles. (**a**) Normalized fluorescence signal at each injection site over time on the left and right side. (**b**) AUCs of ICG clearance curves from left and right flank. Data shown as mean ±S.D. and analyzed by paired Student’s t-test; *p* > 0.05.

Pig and human skin share similarities in structure and thickness (epidermis varying from 30 to 140 μm and 50 to 120 μm, respectively) [39,40,41]. Thus, we decided to perform a pre-clinical validation of the technology, named LymphMonitor 1.0, in vivo in in pig skin. The LymphMeter 1.0 device was used for measuring the fluorescence signal over time. ICG-HSA was injected into the skin of a 1-month-old female domestic pig using MicronJet600^TM^ microneedles (three pyramid-shaped microneedles, each 600 μm long). Injection with microneedles enables standardized and uniform delivery of the tracer formulation through the epidermis directly to the dermal skin layer [36]. The intradermal injections were performed on the left and right flank, and the signals were measured with LymphMeter 1.0 for a total duration of 3 h (every 15 min during the first hour, then every 30 min). This measurement frequency was chosen to enable observation of the initial signal plateau phase due to diffusion of the dye in the tissue and/or distribution to initial lymphatics dye prior to the clearance phase (signal decrease). Figure 3a shows the normalized clearance curves obtained for the left and right flank of the pig. A plateau phase lasting for 30–45 min was observed for 4 out of 6 injections. As fitting using a mono-exponential decay function to calculate half-life would not be appropriate in this case [27], we calculated the area-under-the curve (AUC) to assess the total clearance of the tracer from the injection site. The average AUC calculated for the right side (1.94 ± 0.13) was slightly lower than for the right side (2.29 ± 0.13), however, the difference was not statistically significant (paired Student’s t-test, *p* > 0.05, Figure 3b). As we assume that the skin on both flanks of the pig should be identical in terms of the lymphatic clearance, the slight difference in average AUC on both sides may emerge from not ideally symmetric injections of the tracer. The average pooled AUC was 2.12 ± 0.25. The calculated % Relative Standard Deviation (RSD) for AUC (pooled for right and left flank) was 12%, which demonstrates good repeatability of the measurements. In conclusion, while the mouse experiments confirmed the correct choice of the ICG-HSA concentrations, the pig experiments validated the timeframe (3 h) needed for the measurements using the LymphMeter and confirmed that the lymphatic clearance measurements can be performed with good repeatability.

### 3.3. Human Study

Having validated the appropriate concentrations of ICG and HSA in the solution and the timeframe of the measurements with LymphMeter in the animal studies, we proceeded with the human study to assess whether the method allows for valid assessment of lymphatic function in humans in the context of secondary lymphedema. To this end, we measured the fluorescence signal of intradermally injected ICG-HSA at the injection site in arms or legs of 10 lymphedema patients (diseased extremity versus healthy extremity) over time.

**Figure 4:**
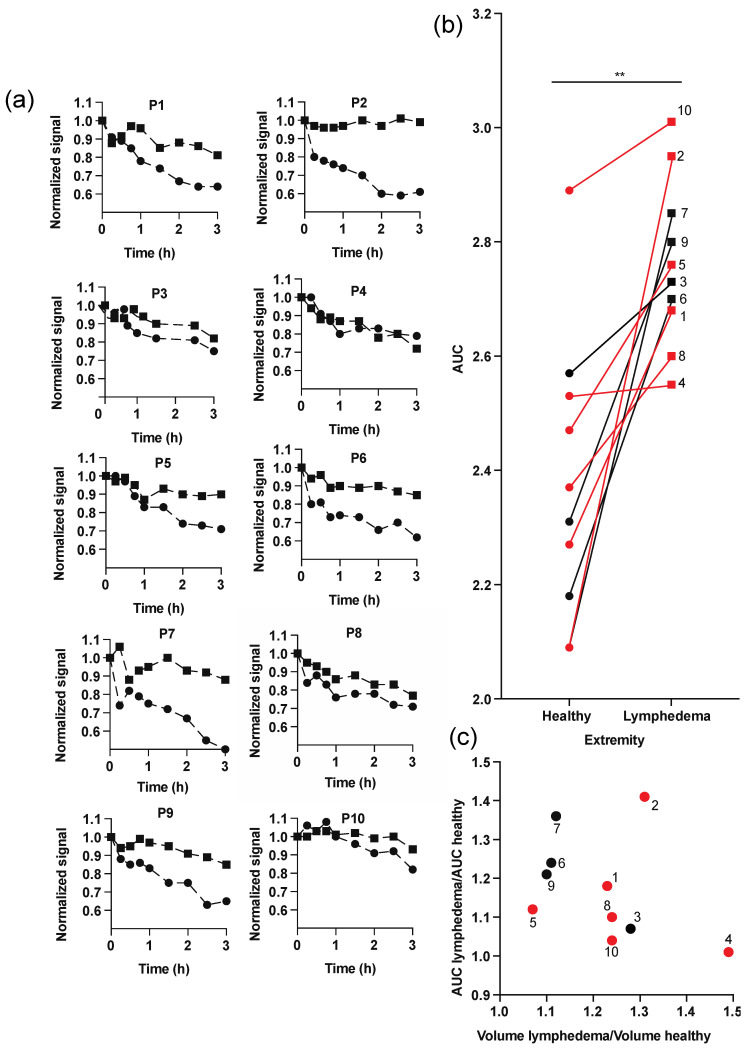
Lymphatic clearance measured by LymphMeter 1.0 in the skin of extremities of 10 patients after intradermal bolus administration of 50 µL of ICG (0.0025 mg/mL) in a solution of 5% HSA using MicronJet600^TM^ microneedles. (**a**) Normalized clearance curves in lymphedematous (square) and contralateral healthy extremity (circle) for each patient. (**b**) AUCs for the lymphedema and the contralateral healthy extremity. Data were compared by Students t-test; ** *p* < 0.01. Data obtained in legs and arms are marked in red and black, respectively. (**c**) Correlation between ratios of AUCs and volumes in healthy and lymphedema extremities. *r* = −0.3647 (Pearson’s correlation coefficient), *p* = 0.3001. Data obtained in legs and arms are marked in red and black, respectively.

Figure 4a shows the normalized clearance curves obtained in each patient. Except for patients 4 and 10, one can clearly appreciate that the signal decrease was more prominent in healthy extremities than in those with lymphedema. The normalized fluorescence signal at 3 h post-injection was 9.5–43.4% higher (average 21.4 ± 12.4%) in lymphedema extremities than in the healthy ones. For some patients, the measured signal varied within 0.5–1 h after the injection (e.g., in patient 1 in the lymphedema extremity and in patient 7 in both extremities). A lack of clear signal decrease immediately after injection may result from diffusion and distribution/redistribution of the dye in the tissue and initial lymphatics prior to the lymphatic clearance phase (signal decrease), which in the case of lymphedema may be longer, due to the increased interstitial fluid accumulation. This phenomenon was already observed in our previous studies [27] in the back skin of mice and in pigs, as well as in several human studies with both radioactive and fluorescent probes [7,18,19,28,42]. As an example, Modi et al. [43] reported an initial increase of the counts of subcutaneously injected radioactive IgG prior to the decrease phase, suggesting transport of the probe to more superficial layers of the skin. Since, due to the initial plateau phase and signal variations, fitting using mono-exponential decay function and half-life calculation was not possible in the clinical study, we used the AUC of the normalized clearance curves to assess the total tissue clearance of the tracer from the injection site. Figure 4b shows the calculated AUCs for the lymphedema and the contralateral healthy extremities. The calculated average AUCs were significantly higher in lymphedema arms and legs (2.76 ± 0.14 h, 95% CI: 2.67–2.85) compared to healthy limbs (2.38 ± 0.25 h, 95% CI: 2.22–2.53), (*p* < 0.01, Student’s t-test). On average, the AUC in lymphedema extremities was 17.3 ± 13.4% (range 0.5–41.0%) higher than in the healthy extremities.

In the past, several studies have used radioactive tracers to investigate their clearance from the injection site after subcutaneous, intramuscular, or intradermal injection. It was found that indeed the clearance was altered in several pathological conditions including lymphedema [42,43,44,45,46,47,48,49,50], and that clearance rates were dependent on the body location [43,51,52] and were changed upon exercise [53]. Other studies using 99mTc-human IgG found that the removal rate constant (k) of intramuscularly and subcutaneously injected tracer was on average 30% lower in the lymphedema forearm compared to the healthy forearm [48,49], and that this reduction was correlated with the degree of swelling [48]. Using the same tracer, another study reported a 46% decrease of the clearance constant ratio (k) in hands swollen due to lymphedema compared to the contralateral non-affected hands after subcutaneous injection [47]. While these studies provided convincing evidence that the depot clearance of radioactive tracers is a useful parameter to quantify lymphatic function, its practical translation to the routine clinic is hampered by the need for using large and stationary gamma cameras as well as radioactivity. By contrast, the LymphMonitor 1.0 technology uses a small, portable detection device, that allows for performing the measurements at any location without the need to expose the patients to ionizing radiation.

We also aimed to establish a correlation of clearance parameters with the extent of the swelling in lymphedema patients. An increase in limb volume is one of the most prominent landmarks of lymphatic insufficiency. In a previous study, a significant correlation of clearance constant (k) ratios and volume ratios of lymphedema and contralateral healthy arms was found [48]. However, we did not find a significant correlation between the AUC ratios (AUC in the lymphedematous limb divided by the AUC of the contralateral limb) and the limb volume ratios (Figure 4c). In patient 4, who had a very prominent swelling of the affected leg (volume ratio 1.49), the AUC ratio was close to 1, whereas in patient 2, with the second largest volume ratio (1.31, leg lymphedema), the AUC ratio was 1.41. This indicates that the limb volume might not be the best predictor of lymphatic function. Interestingly, patient 4 suffered from lymphedema since almost 16 years, whereas patient 2 was diagnosed only two years before the study. An equal AUCs ratio in a patient with long-term, chronic lymphedema may indicate that lymphatic insufficiency may lead to systemic effects and might also influence the contralateral extremity. However, the low number of patients studied and the heterogenous patient population of the present study (patients with leg and arm lymphedema and different etiologies of disease) does not really provide a solid base for investigating such correlations.

The safety and well-being of the patients was monitored throughout the study. In general, the injection of ICG-HSA using MicronJet600^TM^ microneedles was simple, and patients did not report any sign of pain or discomfort during injection. Over the duration of the intervention (3 h), we did not observe any redness or erythema, which demonstrates that the intradermal injections of ICG-HSA are well tolerated.

## 4. Conclusions

Our study provides the first evidence that the quantitative evaluation of lymphatic clearance of intradermally injected ICG-HSA with portable LymphMeter device (LymphMonitor technology) has the potential to evaluate the lymphatic function in patients. There was a significant difference in lymphatic clearance in the lymphedema extremities versus contralateral healthy limbs based on the obtained AUCs of the normalized clearance curves. Thus, the primary objective of the study was achieved, namely the proof-of-concept for the feasibility of the LymphMonitor method to quantitatively assess the lymphatic function in established arm or leg lymphedemas. The present study provides a full overview of the LymphMonitor method development—from the validation of a suitable tracer in mouse studies, through the determination of the required measurement period in pigs to the validation in patients. To the best of our knowledge, this is the first human study showing the use of depot clearance of a fluorescent tracer to quantitatively assess lymphatic function. Currently, we aim to develop a wearable detection device that can be secured to the skin, thus enabling continuous measurements over time. This would eliminate the need for repeated measurements at pre-determined timepoints and minimize the effort of the medical professional who would only need to perform the tracer injection and attach the device to the skin. In the long term, we envisage that patients might be able to perform the functional tests at home, using a self-injection device, and then transmit the results directly to a medical professional using a custom mobile application. This technology would allow consistent monitoring of the lymphatic function in patients after an event that increases the risk of developing lymphedema (e.g. in cancer patients after lymphadenectomy or injury), as well as the progression of the disease, and also to assess the efficiency of applied treatments (e.g., lymphovenous anastomosis surgery). The small and heterogeneous patient cohort constitutes a major limitation of our clinical study. Thus, a more extensive, long-term clinical trial in a larger patient population would help to evaluate the changes in AUC in both lymphedema and healthy extremities throughout the progression of the disease and thus unravel the full potential of the technology for early diagnosis and for monitoring of lymphatic insufficiencies.

## Figures and Tables

**Table 1:** Inclusion and exclusion criteria.

Inclusion Criteria
Females and males 18–75 years old. Established (stage 2 or higher) unilateral secondary arm or leg lymphedema resulting from lymphadectonomy, radiation or any other surgical treatment, infection or injury (classification according to International Society of Lymphology [37]) Good general health status.
**Exclusion Criteria**
Critical illness (active cancer, renal failure, hepatic dysfunction). Active infection. Blood vascular malformations or diseases. Scleroderma. Primary lymphedema. Patients who underwent any surgical procedures for treatment of lymphedema (e.g., lymphovenous anastomosis, liposuction, lymph node transfer). Contradictions to use ICG (VERDYE). Patients with hypersensitivity to ICG or to sodium iodide. Patients with hypersensitivity to iodine. Patients with hyper-thyroidism, patients with autonomic thyroid adenomas. Patients in which the injection of VERDYE was poorly tolerated in the past it must not be used again, since severe anaphylactic reactions might occur. Hypersensitivity to albumin or its excipients. Women, who are pregnant (pregnancy test will be performed in case of women who did not undergo menopause). Women who are breast feeding. Enrolment of the investigator, his/her family members, employees, and other dependent persons. Known or suspected non-compliance, drug or alcohol abuse. Inabilityof the participant to follow the procedures of the study, e.g., due to language problems, psychological disorders, dementia, etc. Participation in another study with an investigational drug within the 30 days preceding and during the present study. Previous enrolment into the current study.

**Table 2:** Study population. Abbreviation: (LEV) lymphedema extremity volume in cm^3^, (HEV) healthy extremity volume in cm^3^, (BMI) body mass index, (FD) First diagnostic.

Patient	Age	Gender	Affected Limb	LEV (cm^3^)	HEV (cm^3^)	Etiology	Clin. Stage	BMI (kg/m^2^)	FD (M/Y)
1	59	F	right leg	23,618	19,212	injury	II	40.1	August 2019
2	63	F	right leg	8670	6613	ovary cancer	II	36.3	November 2018
3	68	F	left arm	3672	2869	breast cancer	II	33.5	July 2017
4	64	F	left leg	11,505	7711	injury	II	22	January 2004
5	40	F	left leg	10,009	9333	melanoma	II	23.8	October 2017
6	61	M	right arm	3158	2852	infection	II	23.5	2008
7	55	M	right arm	3062	2742	melanoma	II	30.3	February 2017
8	49	F	right leg	9565	7735	cervical cancer	II	21	2018
9	48	F	left arm	3049	2765	breast cancer	II	29.1	January 2013
10	48	M	right leg	10,330	8316	melanoma	II	24.5	March 1999

## Supplementary Materials

The following are available online at https://www.mdpi.com/article/10.3390/diagnostics11101873/s1, Supplementary Methods: In vitro fluorescence measurements. Figure S1: Linear relationship between ICG concentrations in purely aqueous solution, 5% and 20% HSA, and fluorescence intensity. ICG concentration range in which the relationship is linear, and respective R^2^ of the fitted regression lines.
