# Supplementary material for: Development and Clinical Validation of the LymphMonitor Technology to Quantitatively Assess Lymphatic Function
Source: Diagnostics (Basel). 2021 Oct 12;11(10):1873. doi: 10.3390/diagnostics11101873 (PMC8534490; doi:10.3390/diagnostics11101873)
Supplement: Supplementary file 1 [file diagnostics-11-01873-s001.zip › diagnostics-1356600-supplementary.pdf]

Supplementary Information

Supplementary Methods:

In vitro fluorescence measurements

The fluorescence intensity of ICG-HSA solutions was measured using plate reader (Tecan Infinite M200 Pro, Tecan GmbH) using Costar 96-well, black, flat-bottom plates (Corning Inc.) Briefly, 200 µl of the solutions were pipetted into the well and the fluorescence intensity was measured using the following settings: λex/λem = 700/750 - 850 nm, gain: 125.

For stability measurements, the fluorescence intensity of the ICG HSA solutions was measured over time for up to 24 h.

Supplementary Figures

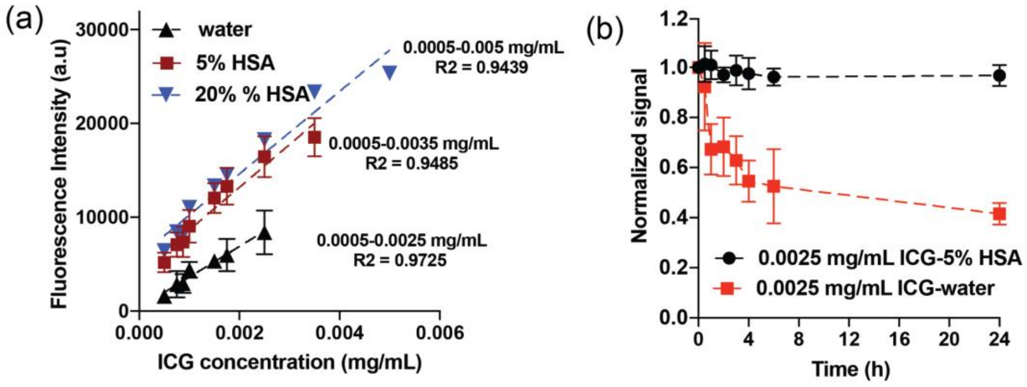

Figure S1. (a) Linear relationship between ICG concentrations in purely aqueous solution, 5% and 20% HSA, and fluorescence intensity (Ex/Em: 770 nm/820 nm). The ICG concentration range in which the relationship is linear, and the respective R^2 of the fitted regression lines are shown. (b) Stability of ICG-water and ICG-5% HSA. Fluorescence intensity of the solutions was measured over 24 h.
